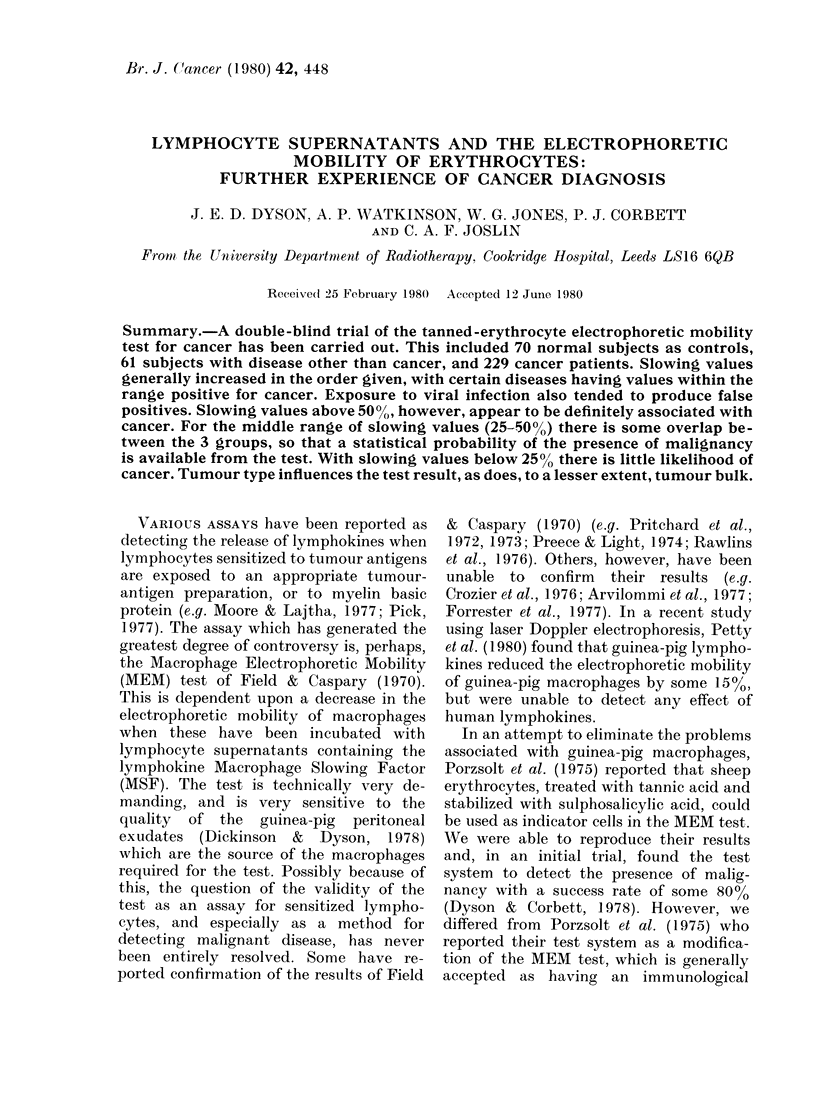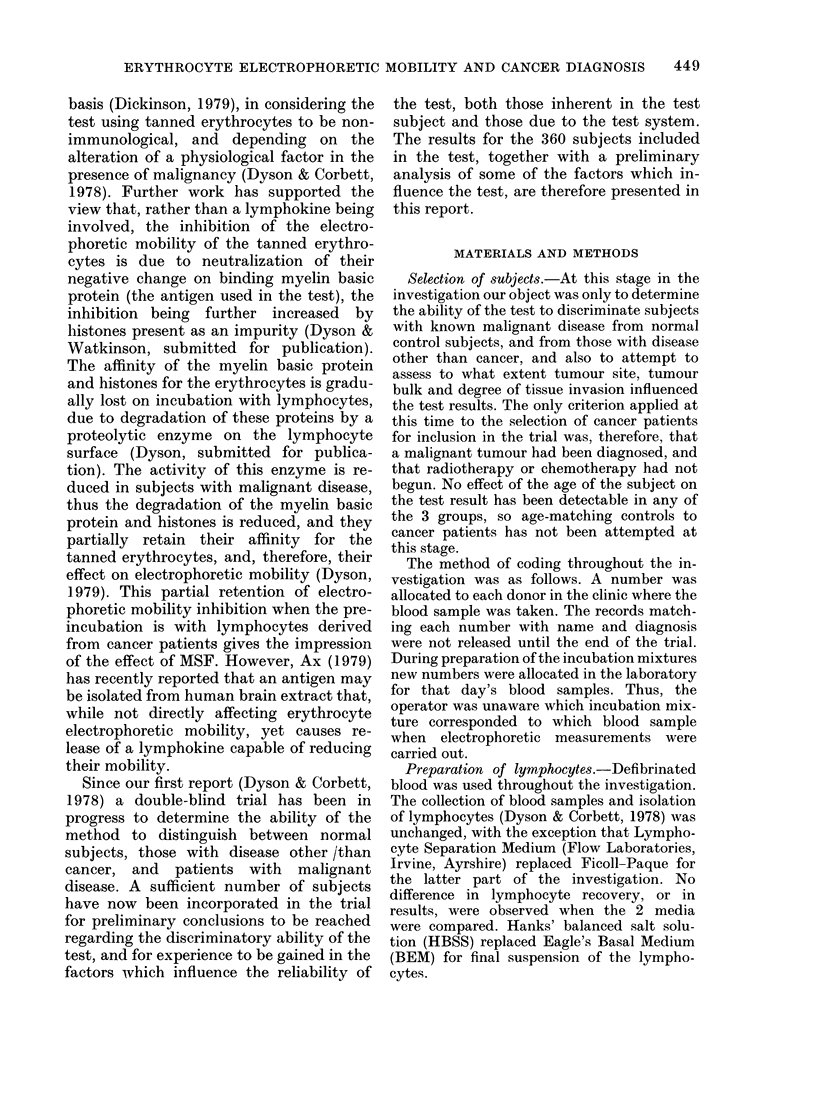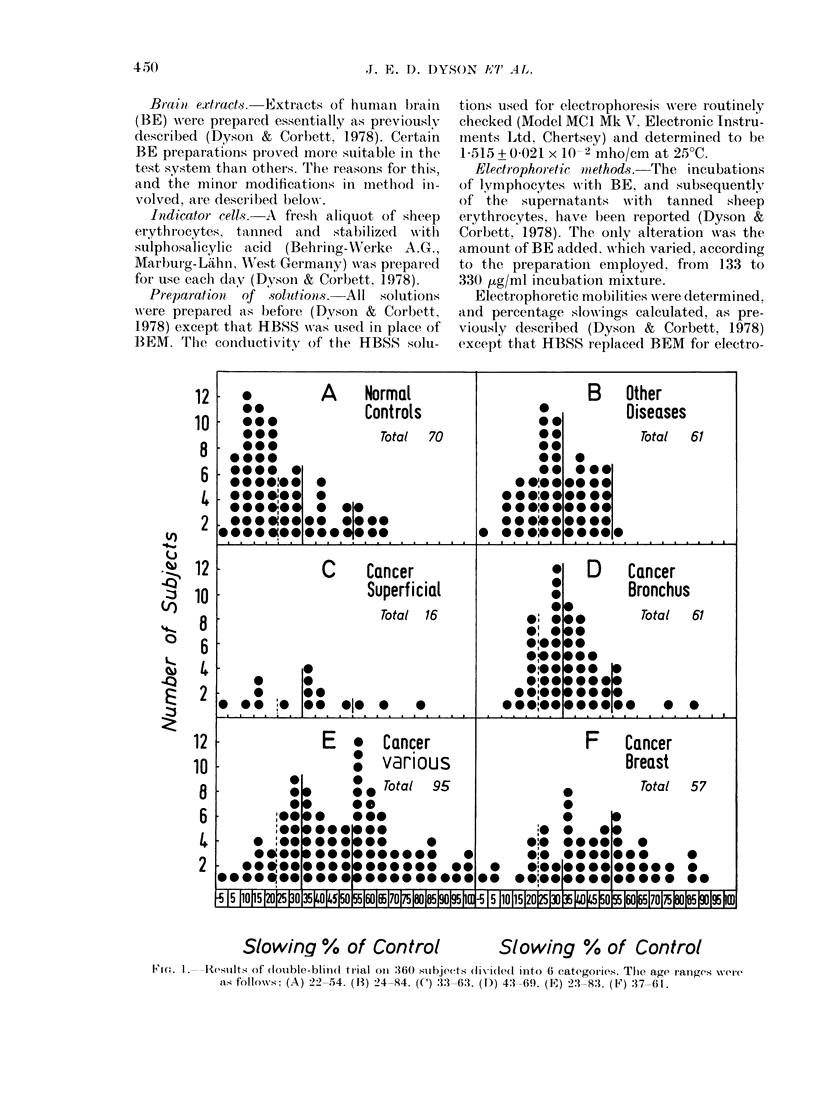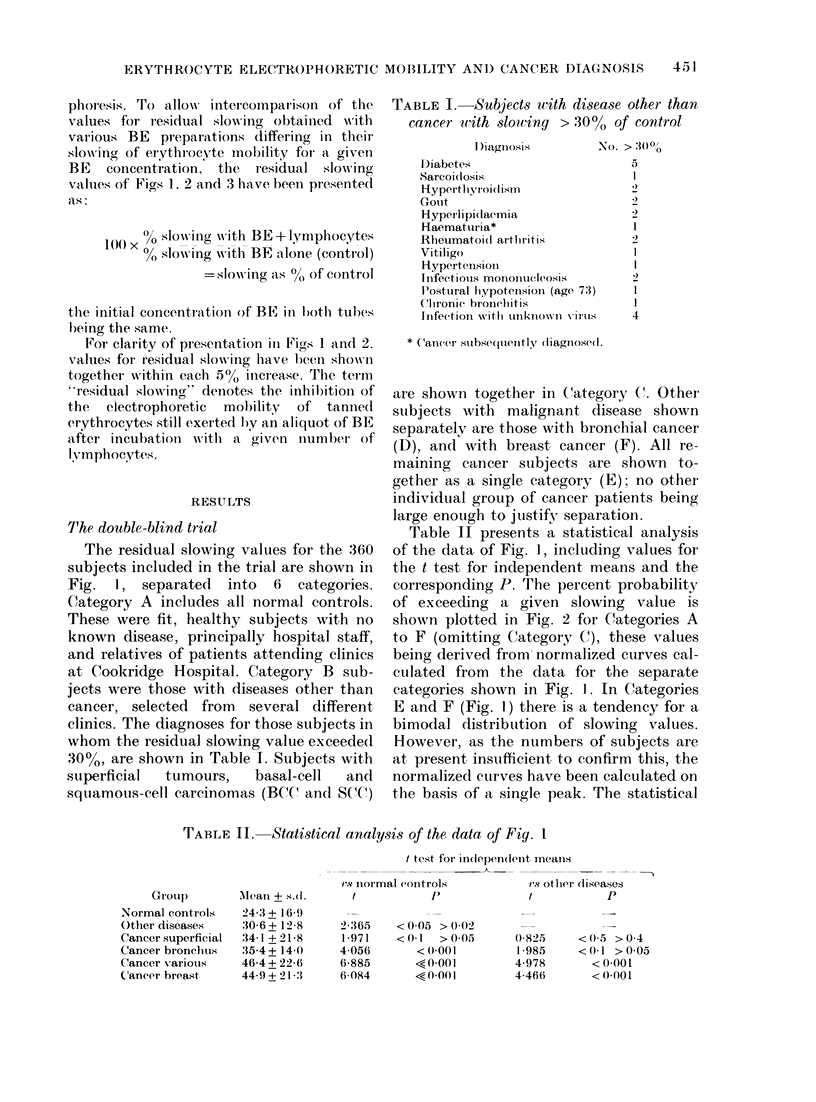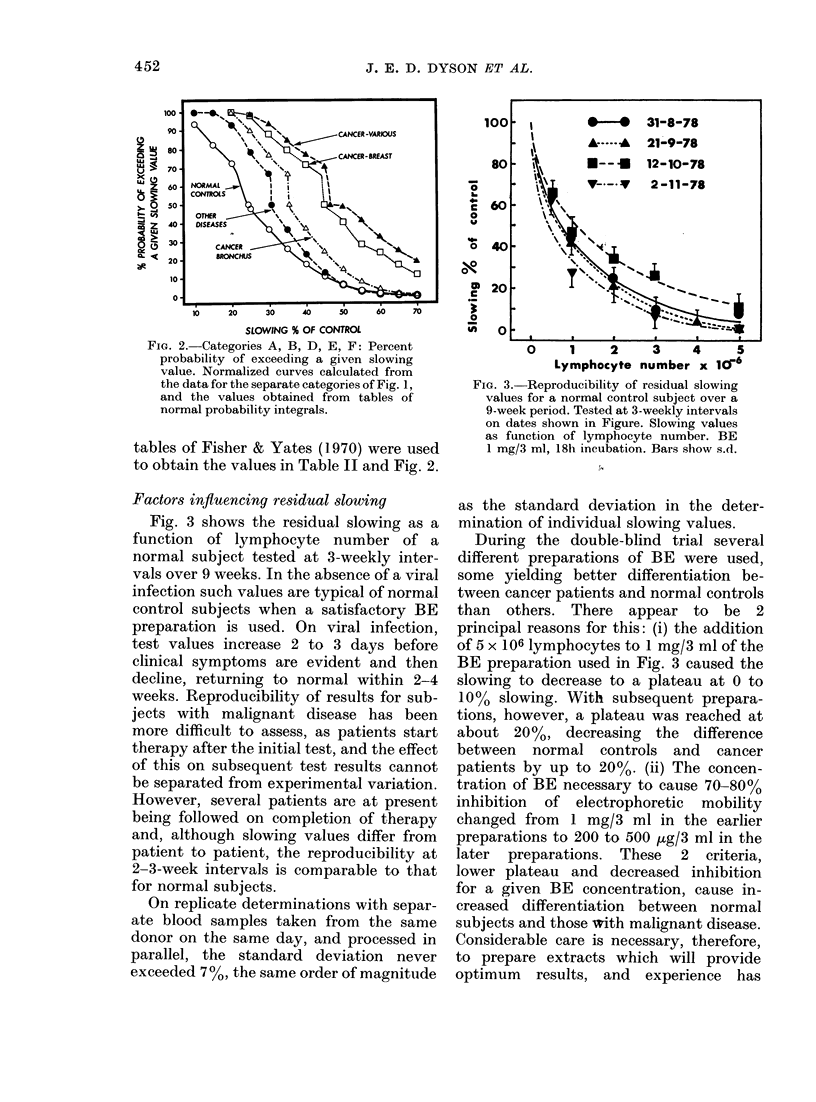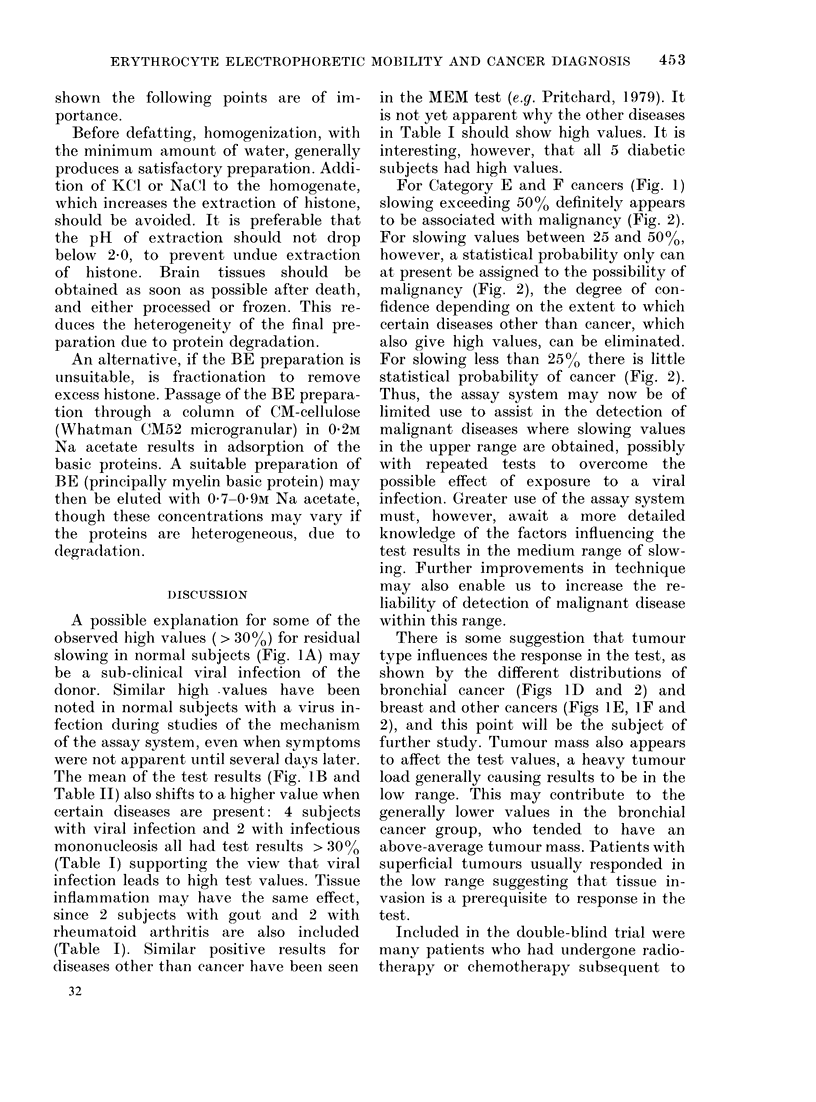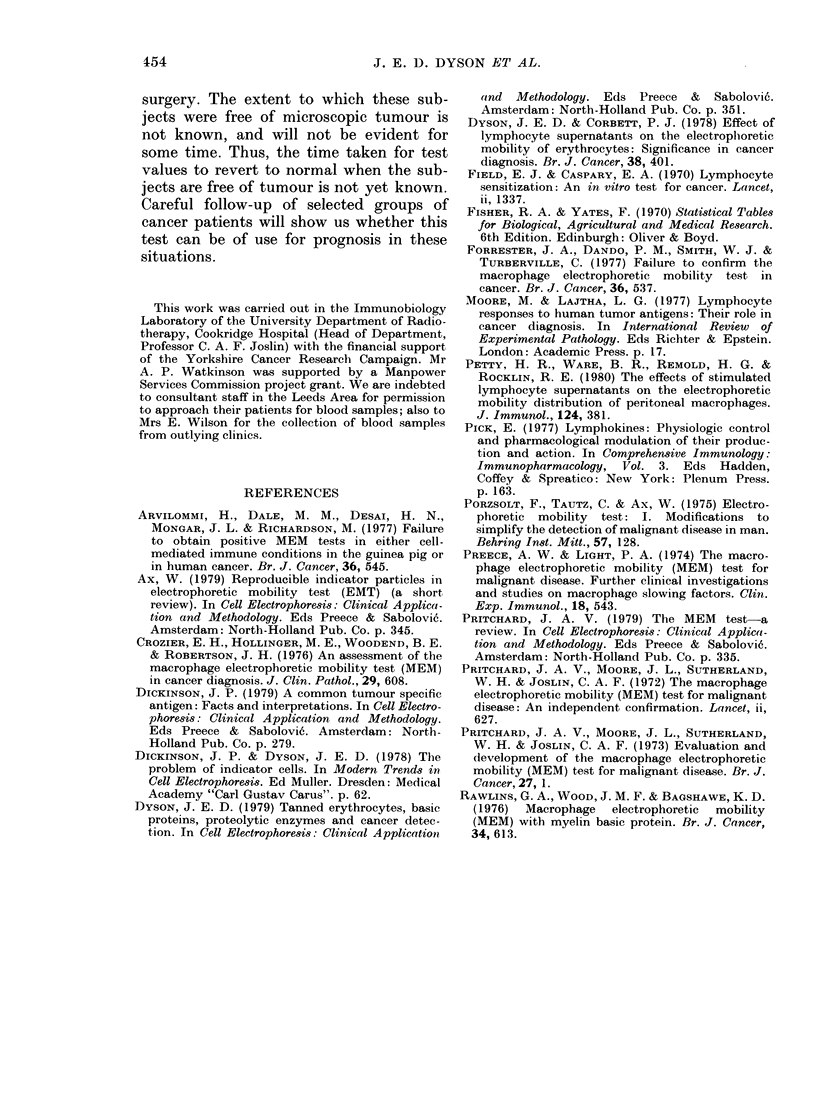# Lymphocyte supernatants and the electrophoretic mobility of erythrocytes: further experience of cancer diagnosis.

**DOI:** 10.1038/bjc.1980.257

**Published:** 1980-09

**Authors:** J. E. Dyson, A. P. Watkinson, W. G. Jones, P. J. Corbett, C. A. Joslin

## Abstract

A double-blind trial of the tanned-erythrocyte electrophoretic mobility test for cancer has been carried out. This included 70 normal subjects as controls, 61 subjects with disease other than cancer, and 229 cancer patients. Slowing values generally increased in the order given, with certain diseases having values within the range positive for cancer. Exposure to viral infection also tended to produce false positives. Slowing values above 50%, however, appear to be definitely associated with cancer. For the middle range of slowing values (25-50%) there is some overlap between the 3 groups, so that a statistical probability of the presence of malignancy is available from the test. With slowing values below 25% there is little likelhood of cancer. Tumour type influences the test result, as does, to a lesser extent, tumour bulk.


Br. J. Cancer (1980) **42**, 448

### LYMPHOCYTE SUPERNATANTS AND THE ELECTROPHORETIC MOBILITY OF ERYTHROCYTES: FURTHER EXPERIENCE OF CANCER DIAGNOSIS

J. E. D. DYSON, A. P. WATKINSON, W. G. JONES, P. J. CORBETT AND C. A. F. JOSLIN

*From the University Department of Radiotherapy, Cookridge Hospital, Leeds LS16 6QB*

Received 25 February 1980 Accepted 12 June 1980

**Summary.—A double-blind trial of the tanned-erythrocyte electrophoretic mobility test for cancer has been carried out. This included 70 normal subjects as controls, 61 subjects with disease other than cancer, and 229 cancer patients. Slowing values generally increased in the order given, with certain diseases having values within the range positive for cancer. Exposure to viral infection also tended to produce false positives. Slowing values above 50%, however, appear to be definitely associated with cancer. For the middle range of slowing values (25–50%) there is some overlap between the 3 groups, so that a statistical probability of the presence of malignancy is available from the test. With slowing values below 25% there is little likelihood of cancer. Tumour type influences the test result, as does, to a lesser extent, tumour bulk.**

VARIOUS ASSAYS have been reported as detecting the release of lymphokines when lymphocytes sensitized to tumour antigens are exposed to an appropriate tumour-antigen preparation, or to myelin basic protein (*e.g.* Moore & Lajtha, 1977; Pick, 1977). The assay which has generated the greatest degree of controversy is, perhaps, the Macrophage Electrophoretic Mobility (MEM) test of Field & Caspary (1970). This is dependent upon a decrease in the electrophoretic mobility of macrophages when these have been incubated with lymphocyte supernatants containing the lymphokine Macrophage Slowing Factor (MSF). The test is technically very demanding, and is very sensitive to the quality of the guinea-pig peritoneal exudates (Dickinson & Dyson, 1978) which are the source of the macrophages required for the test. Possibly because of this, the question of the validity of the test as an assay for sensitized lymphocytes, and especially as a method for detecting malignant disease, has never been entirely resolved. Some have reported confirmation of the results of Field & Caspary (1970) (*e.g.* Pritchard *et al.*, 1972, 1973; Preece & Light, 1974; Rawlins *et al.*, 1976). Others, however, have been unable to confirm their results (*e.g.* Crozier *et al.*, 1976; Arvilommi *et al.*, 1977; Forrester *et al.*, 1977). In a recent study using laser Doppler electrophoresis, Petty *et al.* (1980) found that guinea-pig lymphokines reduced the electrophoretic mobility of guinea-pig macrophages by some 15%, but were unable to detect any effect of human lymphokines.

In an attempt to eliminate the problems associated with guinea-pig macrophages, Porzsolt *et al.* (1975) reported that sheep erythrocytes, treated with tannic acid and stabilized with sulphosalicylic acid, could be used as indicator cells in the MEM test. We were able to reproduce their results and, in an initial trial, found the test system to detect the presence of malignancy with a success rate of some 80% (Dyson & Corbett, 1978). However, we differed from Porzsolt *et al.* (1975) who reported their test system as a modification of the MEM test, which is generally accepted as having an immunological basis (Dickinson, 1979), in considering the test using tanned erythrocytes to be non-immunological, and depending on the alteration of a physiological factor in the presence of malignancy (Dyson & Corbett, 1978). Further work has supported the view that, rather than a lymphokine being involved, the inhibition of the electrophoretic mobility of the tanned erythrocytes is due to neutralization of their negative change on binding myelin basic protein (the antigen used in the test), the inhibition being further increased by histones present as an impurity (Dyson & Watkinson, submitted for publication). The affinity of the myelin basic protein and histones for the erythrocytes is gradually lost on incubation with lymphocytes, due to degradation of these proteins by a proteolytic enzyme on the lymphocyte surface (Dyson, submitted for publication). The activity of this enzyme is reduced in subjects with malignant disease, thus the degradation of the myelin basic protein and histones is reduced, and they partially retain their affinity for the tanned erythrocytes, and, therefore, their effect on electrophoretic mobility (Dyson, 1979). This partial retention of electrophoretic mobility inhibition when the preincubation is with lymphocytes derived from cancer patients gives the impression of the effect of MSF. However, Ax (1979) has recently reported that an antigen may be isolated from human brain extract that, while not directly affecting erythrocyte electrophoretic mobility, yet causes release of a lymphokine capable of reducing their mobility.

Since our first report (Dyson & Corbett, 1978) a double-blind trial has been in progress to determine the ability of the method to distinguish between normal subjects, those with disease other /than cancer, and patients with malignant disease. A sufficient number of subjects have now been incorporated in the trial for preliminary conclusions to be reached regarding the discriminatory ability of the test, and for experience to be gained in the factors which influence the reliability of the test, both those inherent in the test subject and those due to the test system. The results for the 360 subjects included in the test, together with a preliminary analysis of some of the factors which influence the test, are therefore presented in this report.

### MATERIALS AND METHODS

*Selection of subjects.*—At this stage in the investigation our object was only to determine the ability of the test to discriminate subjects with known malignant disease from normal control subjects, and from those with disease other than cancer, and also to attempt to assess to what extent tumour site, tumour bulk and degree of tissue invasion influenced the test results. The only criterion applied at this time to the selection of cancer patients for inclusion in the trial was, therefore, that a malignant tumour had been diagnosed, and that radiotherapy or chemotherapy had not begun. No effect of the age of the subject on the test result has been detectable in any of the 3 groups, so age-matching controls to cancer patients has not been attempted at this stage.

The method of coding throughout the investigation was as follows. A number was allocated to each donor in the clinic where the blood sample was taken. The records matching each number with name and diagnosis were not released until the end of the trial. During preparation of the incubation mixtures new numbers were allocated in the laboratory for that day's blood samples. Thus, the operator was unaware which incubation mixture corresponded to which blood sample when electrophoretic measurements were carried out.

*Preparation of lymphocytes.*—Defibrinated blood was used throughout the investigation. The collection of blood samples and isolation of lymphocytes (Dyson & Corbett, 1978) was unchanged, with the exception that Lymphocyte Separation Medium (Flow Laboratories, Irvine, Ayrshire) replaced Ficoll–Paque for the latter part of the investigation. No difference in lymphocyte recovery, or in results, were observed when the 2 media were compared. Hanks' balanced salt solution (HBSS) replaced Eagle's Basal Medium (BEM) for final suspension of the lymphocytes.

*Brain extracts.*—Extracts of human brain (BE) were prepared essentially as previously described (Dyson & Corbett, 1978). Certain BE preparations proved more suitable in the test system than others. The reasons for this, and the minor modifications in method involved, are described below.

*Indicator cells.*—A fresh aliquot of sheep erythrocytes, tanned and stabilized with sulphosalicylic acid (Behring-Werke A.G., Marburg-Lähn, West Germany) was prepared for use each day (Dyson & Corbett, 1978).

*Preparation of solutions.*—All solutions were prepared as before (Dyson & Corbett, 1978) except that HBSS was used in place of BEM. The conductivity of the HBSS solutions used for electrophoresis were routinely checked (Model MC1 Mk V, Electronic Instruments Ltd, Chertsey) and determined to be $1{\cdot}515 \pm 0{\cdot}021 \times 10^{-2}$ mho/cm at 25°C.

*Electrophoretic methods.*—The incubations of lymphocytes with BE, and subsequently of the supernatants with tanned sheep erythrocytes, have been reported (Dyson & Corbett, 1978). The only alteration was the amount of BE added, which varied, according to the preparation employed, from 133 to 330 $\mu$g/ml incubation mixture.

Electrophoretic mobilities were determined, and percentage slowings calculated, as previously described (Dyson & Corbett, 1978) except that HBSS replaced BEM for electro-

FIG. 1.—Results of double-blind trial on 360 subjects divided into 6 categories. The age ranges were as follows: (A) 22–54. (B) 24–84. (C) 33–63. (D) 43–69. (E) 23–83. (F) 37–61.

phoresis. To allow intercomparison of the values for residual slowing obtained with various BE preparations differing in their slowing of erythrocyte mobility for a given BE concentration, the residual slowing values of Figs 1, 2 and 3 have been presented as:

$$100 \times \frac{\% \text{ slowing with BE} + \text{lymphocytes}}{\% \text{ slowing with BE alone (control)}} = \text{slowing as } \% \text{ of control}$$

the initial concentration of BE in both tubes being the same.

For clarity of presentation in Figs 1 and 2, values for residual slowing have been shown together within each 5% increase. The term "residual slowing" denotes the inhibition of the electrophoretic mobility of tanned erythrocytes still exerted by an aliquot of BE after incubation with a given number of lymphocytes.

### RESULTS

### *The double-blind trial*

The residual slowing values for the 360 subjects included in the trial are shown in Fig. 1, separated into 6 categories. Category A includes all normal controls. These were fit, healthy subjects with no known disease, principally hospital staff, and relatives of patients attending clinics at Cookridge Hospital. Category B subjects were those with diseases other than cancer, selected from several different clinics. The diagnoses for those subjects in whom the residual slowing value exceeded 30%, are shown in Table I. Subjects with superficial tumours, basal-cell and squamous-cell carcinomas (BCC and SCC) are shown together in Category C. Other subjects with malignant disease shown separately are those with bronchial cancer (D), and with breast cancer (F). All remaining cancer subjects are shown together as a single category (E); no other individual group of cancer patients being large enough to justify separation.

Table II presents a statistical analysis of the data of Fig. 1, including values for the *t* test for independent means and the corresponding *P*. The percent probability of exceeding a given slowing value is shown plotted in Fig. 2 for Categories A to F (omitting Category C), these values being derived from normalized curves calculated from the data for the separate categories shown in Fig. 1. In Categories E and F (Fig. 1) there is a tendency for a bimodal distribution of slowing values. However, as the numbers of subjects are at present insufficient to confirm this, the normalized curves have been calculated on the basis of a single peak. The statistical

TABLE I.—*Subjects with disease other than cancer with slowing > 30% of control*

| Diagnosis | No. > 30% |
|---|---|
| Diabetes | 5 |
| Sarcoidosis | 1 |
| Hyperthyroidism | 2 |
| Gout | 2 |
| Hyperlipidaemia | 2 |
| Haematuria* | 1 |
| Rheumatoid arthritis | 2 |
| Vitiligo | 1 |
| Hypertension | 1 |
| Infectious mononucleosis | 2 |
| Postural hypotension (age 73) | 1 |
| Chronic bronchitis | 1 |
| Infection with unknown virus | 4 |

* Cancer subsequently diagnosed.

TABLE II.—*Statistical analysis of the data of Fig. 1*

| | | *t* test for independent means | | | |
|---|---|---|---|---|---|
| | | *vs* normal controls | | *vs* other diseases | |
| Group | Mean ± s.d. | *t* | *P* | *t* | *P* |
| Normal controls | 24·3 ± 16·9 | — | — | — | — |
| Other diseases | 30·6 ± 12·8 | 2·365 | < 0·05 > 0·02 | — | — |
| Cancer superficial | 34·1 ± 21·8 | 1·971 | < 0·1 > 0·05 | 0·825 | < 0·5 > 0·4 |
| Cancer bronchus | 35·4 ± 14·0 | 4·056 | < 0·001 | 1·985 | < 0·1 > 0·05 |
| Cancer various | 46·4 ± 22·6 | 6·885 | ≪ 0·001 | 4·978 | < 0·001 |
| Cancer breast | 44·9 ± 21·3 | 6·084 | ≪ 0·001 | 4·466 | < 0·001 |

FIG. 2.—Categories A, B, D, E, F: Percent probability of exceeding a given slowing value. Normalized curves calculated from the data for the separate categories of Fig. 1, and the values obtained from tables of normal probability integrals.

FIG. 3.—Reproducibility of residual slowing values for a normal control subject over a 9-week period. Tested at 3-weekly intervals on dates shown in Figure. Slowing values as function of lymphocyte number. BE 1 mg/3 ml, 18h incubation. Bars show s.d.

tables of Fisher & Yates (1970) were used to obtain the values in Table II and Fig. 2.

*Factors influencing residual slowing*

Fig. 3 shows the residual slowing as a function of lymphocyte number of a normal subject tested at 3-weekly intervals over 9 weeks. In the absence of a viral infection such values are typical of normal control subjects when a satisfactory BE preparation is used. On viral infection, test values increase 2 to 3 days before clinical symptoms are evident and then decline, returning to normal within 2–4 weeks. Reproducibility of results for subjects with malignant disease has been more difficult to assess, as patients start therapy after the initial test, and the effect of this on subsequent test results cannot be separated from experimental variation. However, several patients are at present being followed on completion of therapy and, although slowing values differ from patient to patient, the reproducibility at 2–3-week intervals is comparable to that for normal subjects.

On replicate determinations with separate blood samples taken from the same donor on the same day, and processed in parallel, the standard deviation never exceeded 7%, the same order of magnitude as the standard deviation in the determination of individual slowing values.

During the double-blind trial several different preparations of BE were used, some yielding better differentiation between cancer patients and normal controls than others. There appear to be 2 principal reasons for this: (i) the addition of $5 \times 10^6$ lymphocytes to 1 mg/3 ml of the BE preparation used in Fig. 3 caused the slowing to decrease to a plateau at 0 to 10% slowing. With subsequent preparations, however, a plateau was reached at about 20%, decreasing the difference between normal controls and cancer patients by up to 20%. (ii) The concentration of BE necessary to cause 70–80% inhibition of electrophoretic mobility changed from 1 mg/3 ml in the earlier preparations to 200 to 500 $\mu$g/3 ml in the later preparations. These 2 criteria, lower plateau and decreased inhibition for a given BE concentration, cause increased differentiation between normal subjects and those with malignant disease. Considerable care is necessary, therefore, to prepare extracts which will provide optimum results, and experience has shown the following points are of importance.

Before defatting, homogenization, with the minimum amount of water, generally produces a satisfactory preparation. Addition of KCl or NaCl to the homogenate, which increases the extraction of histone, should be avoided. It is preferable that the pH of extraction should not drop below 2·0, to prevent undue extraction of histone. Brain tissues should be obtained as soon as possible after death, and either processed or frozen. This reduces the heterogeneity of the final preparation due to protein degradation.

An alternative, if the BE preparation is unsuitable, is fractionation to remove excess histone. Passage of the BE preparation through a column of CM-cellulose (Whatman CM52 microgranular) in 0·2M Na acetate results in adsorption of the basic proteins. A suitable preparation of BE (principally myelin basic protein) may then be eluted with 0·7–0·9M Na acetate, though these concentrations may vary if the proteins are heterogeneous, due to degradation.

### DISCUSSION

A possible explanation for some of the observed high values (> 30%) for residual slowing in normal subjects (Fig. 1A) may be a sub-clinical viral infection of the donor. Similar high values have been noted in normal subjects with a virus infection during studies of the mechanism of the assay system, even when symptoms were not apparent until several days later. The mean of the test results (Fig. 1B and Table II) also shifts to a higher value when certain diseases are present: 4 subjects with viral infection and 2 with infectious mononucleosis all had test results > 30% (Table I) supporting the view that viral infection leads to high test values. Tissue inflammation may have the same effect, since 2 subjects with gout and 2 with rheumatoid arthritis are also included (Table I). Similar positive results for diseases other than cancer have been seen in the MEM test (*e.g.* Pritchard, 1979). It is not yet apparent why the other diseases in Table I should show high values. It is interesting, however, that all 5 diabetic subjects had high values.

For Category E and F cancers (Fig. 1) slowing exceeding 50% definitely appears to be associated with malignancy (Fig. 2). For slowing values between 25 and 50%, however, a statistical probability only can at present be assigned to the possibility of malignancy (Fig. 2), the degree of confidence depending on the extent to which certain diseases other than cancer, which also give high values, can be eliminated. For slowing less than 25% there is little statistical probability of cancer (Fig. 2). Thus, the assay system may now be of limited use to assist in the detection of malignant diseases where slowing values in the upper range are obtained, possibly with repeated tests to overcome the possible effect of exposure to a viral infection. Greater use of the assay system must, however, await a more detailed knowledge of the factors influencing the test results in the medium range of slowing. Further improvements in technique may also enable us to increase the reliability of detection of malignant disease within this range.

There is some suggestion that tumour type influences the response in the test, as shown by the different distributions of bronchial cancer (Figs 1D and 2) and breast and other cancers (Figs 1E, 1F and 2), and this point will be the subject of further study. Tumour mass also appears to affect the test values, a heavy tumour load generally causing results to be in the low range. This may contribute to the generally lower values in the bronchial cancer group, who tended to have an above-average tumour mass. Patients with superficial tumours usually responded in the low range suggesting that tissue invasion is a prerequisite to response in the test.

Included in the double-blind trial were many patients who had undergone radiotherapy or chemotherapy subsequent to surgery. The extent to which these subjects were free of microscopic tumour is not known, and will not be evident for some time. Thus, the time taken for test values to revert to normal when the subjects are free of tumour is not yet known. Careful follow-up of selected groups of cancer patients will show us whether this test can be of use for prognosis in these situations.

This work was carried out in the Immunobiology Laboratory of the University Department of Radiotherapy, Cookridge Hospital (Head of Department, Professor C. A. F. Joslin) with the financial support of the Yorkshire Cancer Research Campaign. Mr A. P. Watkinson was supported by a Manpower Services Commission project grant. We are indebted to consultant staff in the Leeds Area for permission to approach their patients for blood samples; also to Mrs E. Wilson for the collection of blood samples from outlying clinics.

### REFERENCES

ARVILOMMI, H., DALE, M. M., DESAI, H. N., MONGAR, J. L. & RICHARDSON, M. (1977) Failure to obtain positive MEM tests in either cell-mediated immune conditions in the guinea pig or in human cancer. *Br. J. Cancer*, **36**, 545.

AX, W. (1979) Reproducible indicator particles in electrophoretic mobility test (EMT) (a short review). In *Cell Electrophoresis: Clinical Application and Methodology*. Eds Preece & Sabolović. Amsterdam: North-Holland Pub. Co. p. 345.

CROZIER, E. H., HOLLINGER, M. E., WOODEND, B. E. & ROBERTSON, J. H. (1976) An assessment of the macrophage electrophoretic mobility test (MEM) in cancer diagnosis. *J. Clin. Pathol.*, **29**, 608.

DICKINSON, J. P. (1979) A common tumour specific antigen: Facts and interpretations. In *Cell Electrophoresis: Clinical Application and Methodology*. Eds Preece & Sabolović. Amsterdam: North-Holland Pub. Co. p. 279.

DICKINSON, J. P. & DYSON, J. E. D. (1978) The problem of indicator cells. In *Modern Trends in Cell Electrophoresis*. Ed Muller. Dresden: Medical Academy "Carl Gustav Carus". p. 62.

DYSON, J. E. D. (1979) Tanned erythrocytes, basic proteins, proteolytic enzymes and cancer detection. In *Cell Electrophoresis: Clinical Application and Methodology*. Eds Preece & Sabolović. Amsterdam: North-Holland Pub. Co. p. 351.

DYSON, J. E. D. & CORBETT, P. J. (1978) Effect of lymphocyte supernatants on the electrophoretic mobility of erythrocytes: Significance in cancer diagnosis. *Br. J. Cancer*, **38**, 401.

FIELD, E. J. & CASPARY, E. A. (1970) Lymphocyte sensitization: An *in vitro* test for cancer. *Lancet*, ii, 1337.

FISHER, R. A. & YATES, F. (1970) *Statistical Tables for Biological, Agricultural and Medical Research*. 6th Edition. Edinburgh: Oliver & Boyd.

FORRESTER, J. A., DANDO, P. M., SMITH, W. J. & TURBERVILLE, C. (1977) Failure to confirm the macrophage electrophoretic mobility test in cancer. *Br. J. Cancer*, **36**, 537.

MOORE, M. & LAJTHA, L. G. (1977) Lymphocyte responses to human tumor antigens: Their role in cancer diagnosis. In *International Review of Experimental Pathology*. Eds Richter & Epstein. London: Academic Press. p. 17.

PETTY, H. R., WARE, B. R., REMOLD, H. G. & ROCKLIN, R. E. (1980) The effects of stimulated lymphocyte supernatants on the electrophoretic mobility distribution of peritoneal macrophages. *J. Immunol.*, **124**, 381.

PICK, E. (1977) Lymphokines: Physiologic control and pharmacological modulation of their production and action. In *Comprehensive Immunology: Immunopharmacology, Vol.* 3. Eds Hadden, Coffey & Spreatico: New York: Plenum Press. p. 163.

PORZSOLT, F., TAUTZ, C. & AX, W. (1975) Electrophoretic mobility test: I. Modifications to simplify the detection of malignant disease in man. *Behring Inst. Mitt.*, **57**, 128.

PREECE, A. W. & LIGHT, P. A. (1974) The macrophage electrophoretic mobility (MEM) test for malignant disease. Further clinical investigations and studies on macrophage slowing factors. *Clin. Exp. Immunol.*, **18**, 543.

PRITCHARD, J. A. V. (1979) The MEM test—a review. In *Cell Electrophoresis: Clinical Application and Methodology*. Eds Preece & Sabolović. Amsterdam: North-Holland Pub. Co. p. 335.

PRITCHARD, J. A. V., MOORE, J. L., SUTHERLAND, W. H. & JOSLIN, C. A. F. (1972) The macrophage electrophoretic mobility (MEM) test for malignant disease: An independent confirmation. *Lancet*, ii, 627.

PRITCHARD, J. A. V., MOORE, J. L., SUTHERLAND, W. H. & JOSLIN, C. A. F. (1973) Evaluation and development of the macrophage electrophoretic mobility (MEM) test for malignant disease. *Br. J. Cancer*, **27**, 1.

RAWLINS, G. A., WOOD, J. M. F. & BAGSHAWE, K. D. (1976) Macrophage electrophoretic mobility (MEM) with myelin basic protein. *Br. J. Cancer*, **34**, 613.